# Open Peri-Implant Distal Radius Fracture Following Previous Treatment With a Dorsal Nail-Plate Device: A Case Report

**DOI:** 10.7759/cureus.89930

**Published:** 2025-08-12

**Authors:** Abdulai Bangura, Mubinah Khaleel, Lasun Oladeji, Douglas Haase, Kyle Schweser

**Affiliations:** 1 Department of Orthopedic Surgery, University of Missouri School of Medicine, Columbia, USA

**Keywords:** distal radius fracture, implant-related fracture, open ulna fracture, peri-implant fracture, radius intramedullary nail

## Abstract

Peri-implant distal radius fractures are rare but are expected to increase in incidence due to the shift from closed reduction and casting to operative fixation. The literature on distal radius peri-implant fractures is limited, with nearly all reported cases involving volar plate osteosynthesis. We report a case of an adult male who sustained a peri-implant distal radius fracture around a previously placed dorsal intramedullary nail-plate device, accompanied by an open ulnar fracture. A healthy 36-year-old right-hand-dominant male experienced this injury following a fall from a motorized scooter. During surgery, the deformity of the intramedullary device prevented fracture reduction. The device was cut, and open reduction and internal fixation were then performed using a unique approach. No complications occurred, and the patient returned to all daily activities three months after surgery. With increasing rates of operative fixation, the prevalence of peri-implant distal radius fractures is likely to rise. This case highlights the management of a peri-implant fracture involving a dorsal intramedullary nail-plate device, a scenario not previously described in the literature, and underscores the need for a tailored surgical approach.

## Introduction

Distal radius fractures are among the most common fractures in adults and account for one-third of all fractures in the elderly [1]. Treatment of these injuries has evolved from closed reduction and casting to operative fixation for displaced fractures [2]. Although operative fixation has been shown to improve early function, radiographic outcomes, and grip strength, it is associated with increased complication rates [3-8]. The literature reports a wide range of complication rates (6-80%), including tendon injuries, infections, malunion, and complex regional pain syndrome [9]. Despite the frequency of complications, literature on distal radius peri-implant fractures remains limited, with nearly all reported cases involving volar plate osteosynthesis [10-14]. We report a case of an adult male who sustained a peri-implant distal radius fracture around a previously placed dorsal intramedullary nail-plate (Hand Innovations, LLC, Miami, FL, USA) with a concomitant open ulnar fracture.

## Case presentation

A 36-year-old otherwise healthy right-hand-dominant male was evaluated in our emergency department following a fall from a motorized scooter. His surgical history included operative fixation of a left distal radius fracture with a dorsal intramedullary nail-plate 16 years earlier at an outside facility. He reported daily tobacco and marijuana use.

**Figure 1:**
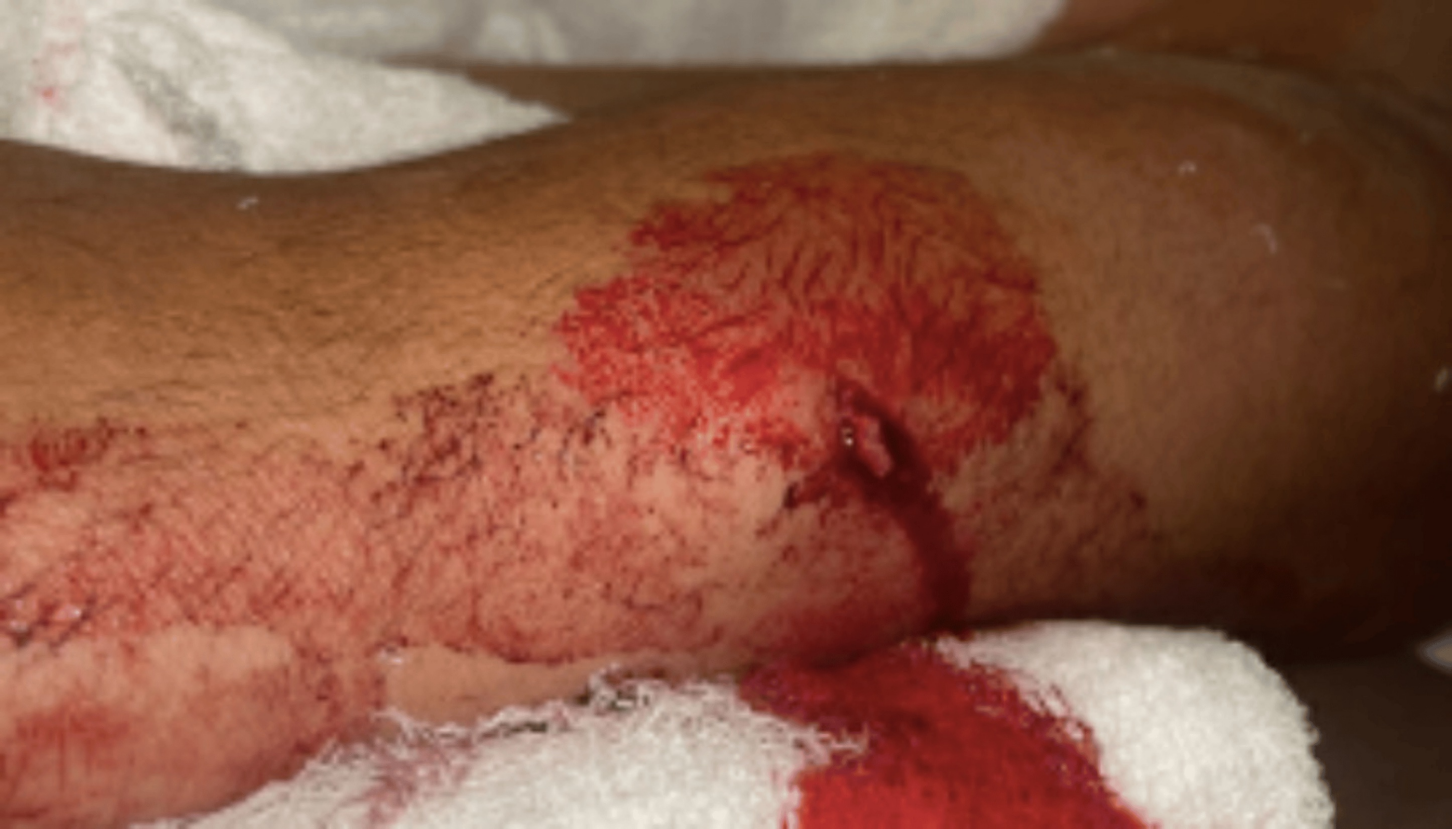
Left forearm showing a 1 to 2 cm laceration on the ulnar aspect of the distal forearm, with the wound tracking down to the bone

**Figure 2:**
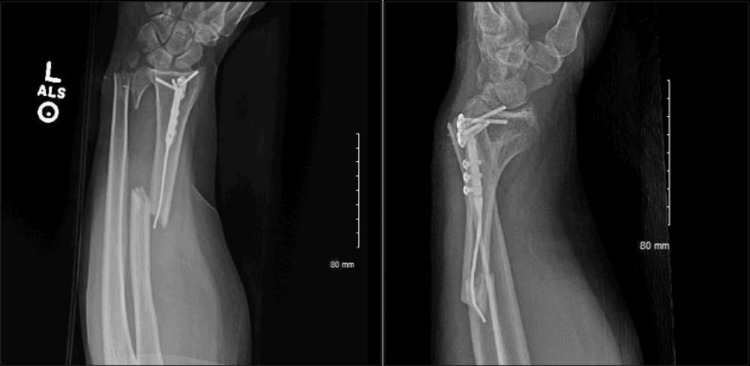
Anteroposterior (A) and lateral (B) X-ray films demonstrating an extra-articular peri-implant fracture of the left distal radius, along with a concomitant displaced intra-articular comminuted fracture of the distal ulna

On physical examination, there was a 1 to 2 cm laceration on the ulnar aspect of the left distal forearm, consistent with an open fracture. The left distal forearm was grossly edematous without obvious deformity (Figure 1). Wrist range of motion was limited due to pain, and no neurological or vascular deficits were noted. Radiographs revealed an extraarticular peri-implant fracture of the distal radius with 100% displacement, 8 mm shortening, and 15-degree volar angulation. Additionally, there was a displaced intraarticular comminuted fracture of the distal ulna with an overlying soft tissue defect (Figure 2a, 2b).

The patient was initially managed with bedside irrigation, reduction, and application of a sugar-tong splint. He received Ancef, and his tetanus vaccination was updated in the emergency room. The following day, the patient was taken to the operating room for irrigation and debridement, followed by open reduction and internal fixation of both fractures, performed by the senior author.

A standard volar Henry approach was utilized. The radius fracture was identified, with the proximal portion of the nail-plate device deformed volarly and protruding from the distal fragment. The protruding segment of the device prevented reduction of the radius despite attempts to correct the device’s deformity, so it was cut short with a wire cutter. The peri-implant radius fracture was then debrided, irrigated, and reduced. Once reduction was achieved, a 2.7 mm lag screw was placed. Subsequently, a nine-hole 3.5 mm limited-contact dynamic compression plate was applied to the radius to achieve adequate compression.

**Figure 3:**
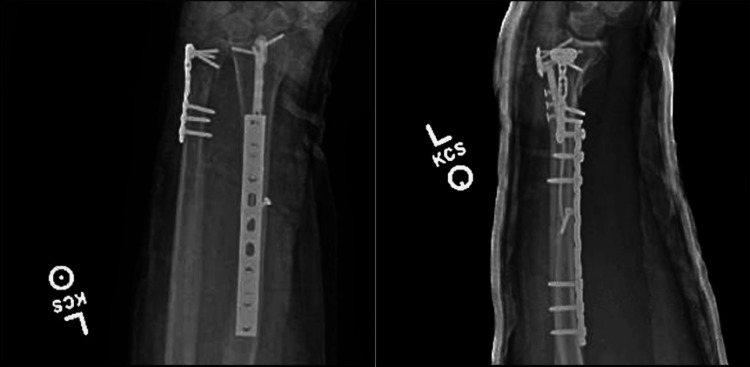
Anteroposterior (A) and lateral (B) X-ray films demonstrating restoration of anatomic alignment of the peri-implant fracture of the left distal radius using a 2.7 mm lag screw and a nine-hole 3.5 mm limited-contact dynamic compression plate The left distal ulna fracture was restored with a contoured 2.7 mm T-plate. A sugar-tong splint is in place.

The traumatic wound was extended to expose the ulna fracture, which was thoroughly irrigated and debrided. After fracture reduction, fixation was achieved using a contoured 2.7 mm T-plate. Before closing the surgical wounds, the wrist and forearm were taken through a full range of motion without impingement or signs of instability, including stability of the distal radioulnar joint. Final imaging confirmed restoration of anatomic alignment (Figure 3a, 3b).

**Figure 4:**
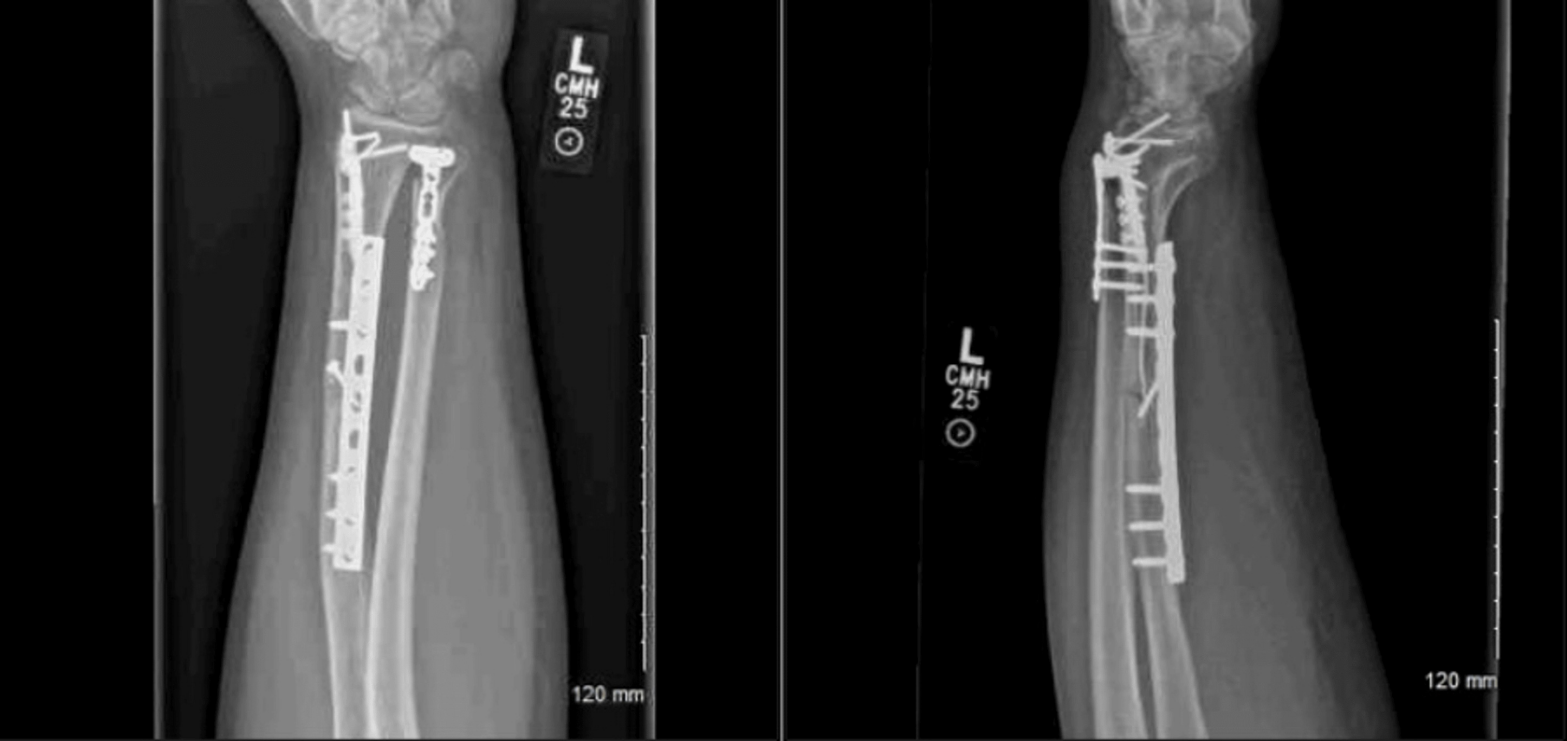
Three-month postoperative anteroposterior (A) and lateral (B) X-ray films demonstrating intact hardware and bone callus formation at the fracture sites

Postoperatively, the patient was placed in a sugar-tong splint for two weeks due to the comminuted ulna fracture and distal radioulnar joint involvement before transitioning to a cock-up wrist splint. At that time, rehabilitation was initiated to improve the range of motion. After three months, the patient achieved full, painless flexion and extension of the wrist, with 80 degrees of supination and pronation, although mild discomfort remained at terminal supination. Follow-up X-rays showed intact hardware and bone callus formation over the fracture sites (Figure 4a, 4b). No complications were encountered, and the patient returned to all daily activities performed prior to the injury.

## Discussion

Although peri-implant distal radius fractures are rare, their incidence is expected to increase due to the shift from closed reduction and casting to operative fixation [10]. As more cases are reported, authors have begun proposing classification systems and treatment strategies; however, none have yet gained widespread acceptance [1,15-18]. Research remains insufficient to fully understand the risk factors for these fractures (including implant type), fracture subtypes, and optimal management based on subtype.

In contrast to previously reported peri-implant distal radius fractures, which almost exclusively involve volar plate implants [10-14], our case involved a dorsal intramedullary nail-plate device. Over recent decades, volar locking plates have gained popularity, as dorsal locking nail-plates have been associated with extensor tendon complications such as tenosynovitis and tendon rupture. To reduce hardware prominence and tendon irritation, unique dorsal plate designs like the dorsal intramedullary nail-plate used in our case were developed. However, literature remains scarce regarding which implant types carry a higher risk of peri-implant fracture.

Fractures around distal radius implants can occur in various patterns. Kistler et al. classified peri-implant fractures using the Unified Classification System for Periprosthetic Fractures, though it was not originally intended for internal fixation devices [10]. Some authors have applied newly proposed classifications [11,13], while others have avoided classification altogether [12,14]. We used the classification system by Egol et al., which demonstrated significant interobserver reliability (k = 0.839, p < 0.0005). According to their system, our case represents a type II peri-implant fracture, occurring between the most proximal or distal screw and the corresponding end of the plate or implant. This type accounted for two-thirds of all peri-implant fractures in their cohort of 103 cases [16].

Management of our case required cutting a portion of the fixed implant to restore anatomic alignment, a surgical approach not previously described in the literature. The absence of established and widely accepted surgical guidelines can make treating distal radius peri-implant fractures challenging.

## Conclusions

The literature on peri-implant distal radius fractures remains limited, complicating their management. Our case involved a peri-implant distal radius fracture around a previously placed dorsal intramedullary nail-plate device with a concomitant open ulnar fracture. Management of this type of peri-implant fracture had not been previously described and required a tailored approach. Further research is necessary to better understand fracture risks (including implant type), subtypes, and subtype-specific management strategies for peri-implant distal radius fractures.
